# Supplementary material for: Continental shelves as a variable but increasing global sink for atmospheric carbon dioxide
Source: Nat Commun. 2018 Jan 31;9:454. doi: 10.1038/s41467-017-02738-z (PMC5792465; doi:10.1038/s41467-017-02738-z)
Supplement: Supplementary file 1 — Supplementary Information [file 41467_2017_2738_MOESM1_ESM.pdf]

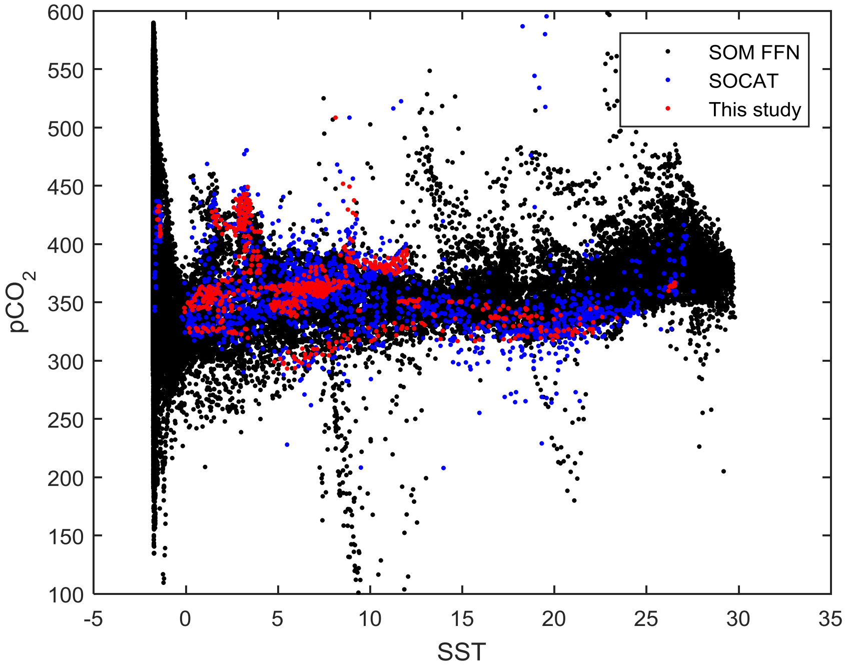

Supplementary Figure 1: Distribution of pairs of winter pCO_2 and SST for all cells extracted from the SOM_FFN data product of Laruelle and co-authors[1] (black dots), the SOCAT database (blue dots) and the 825 cells for which trends in d$\Delta p CO_2$/dt were calculated in this study (red dots).

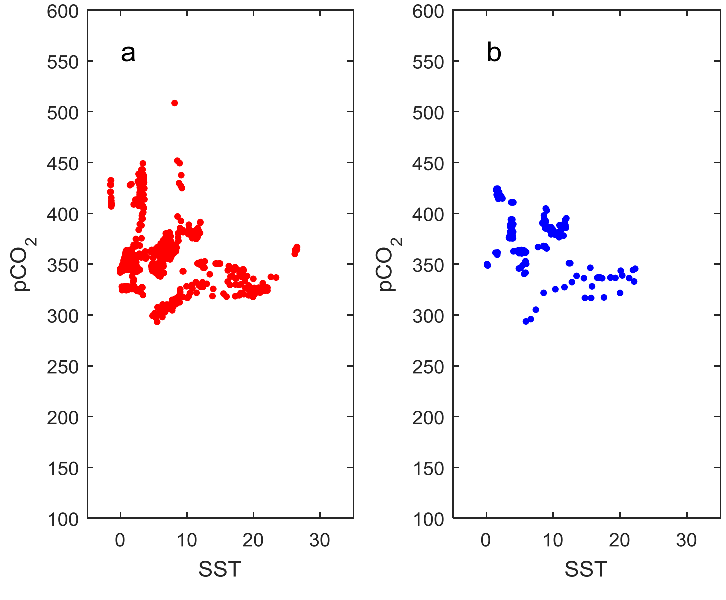

Supplementary Figure 2: Distribution of pairs of winter pCO_2 and SST for cells with d$\Delta p CO_2$/dt>0 (a, red) and d$\Delta p CO_2$/dt<0 (b, blue).

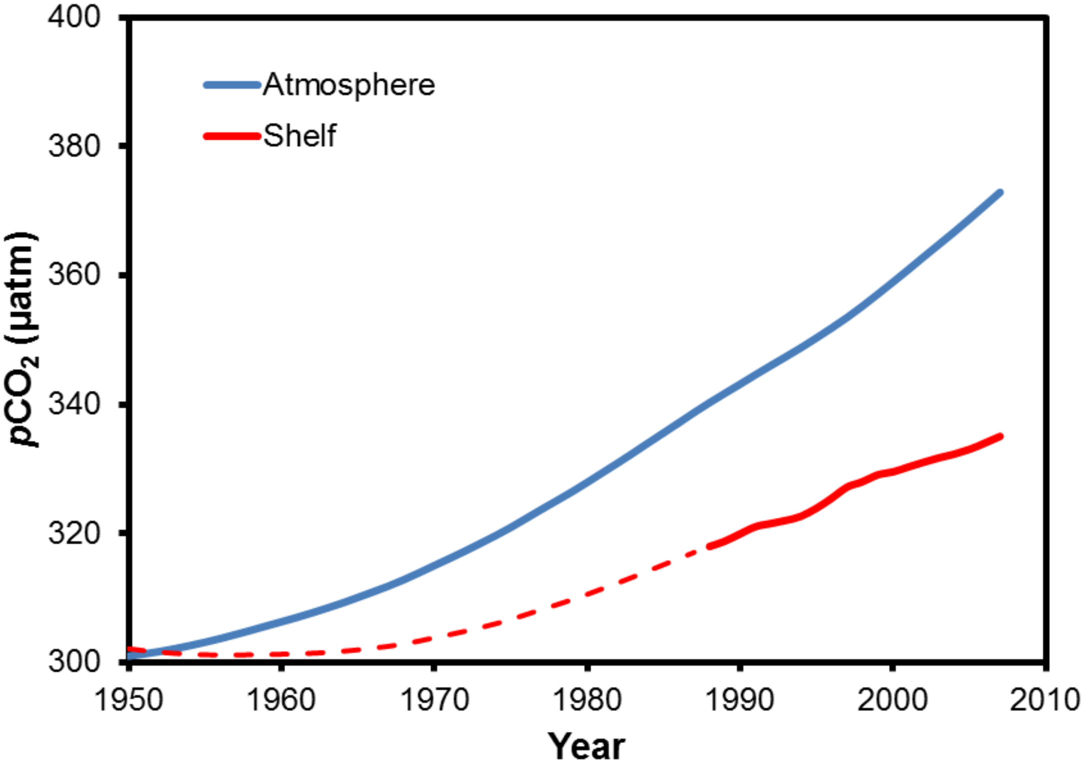

Supplementary Figure 3: Evolution of atmospheric (blue) and shelf pCO_2 (red) over the 1950-2010 period. The continuous line correspond to the period for which the trend is derived from the SOCAT data while the dashed line correspond to an extrapolation of the rate of $d(\Delta pCO_2)/dt$ from the 1988-1993 period to earlier years.

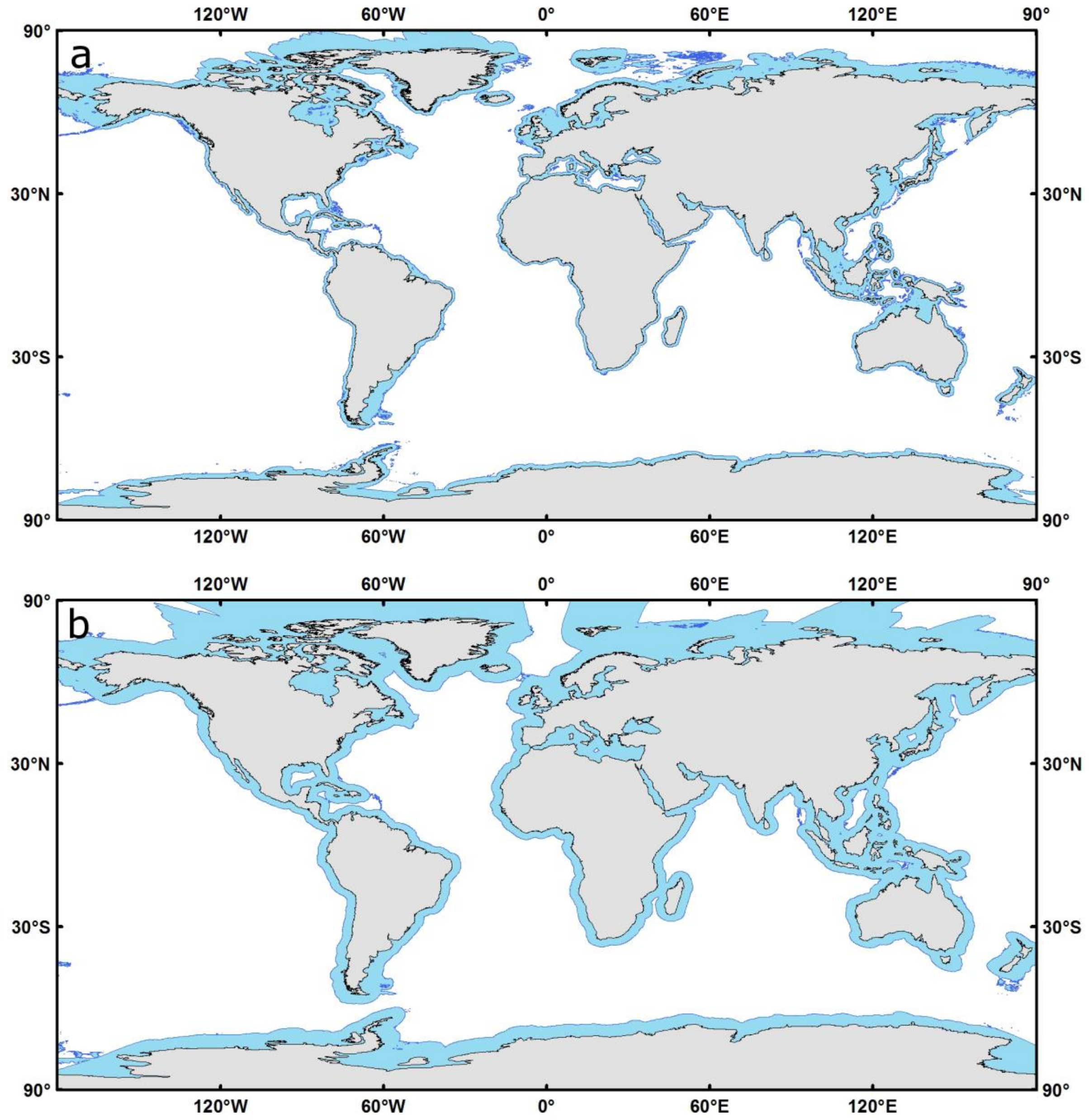

Supplementary Figure 4: Limits of the coastal ocean using 200m water depth (a) or using 500m water depth or 100km distance from the shoreline (b).

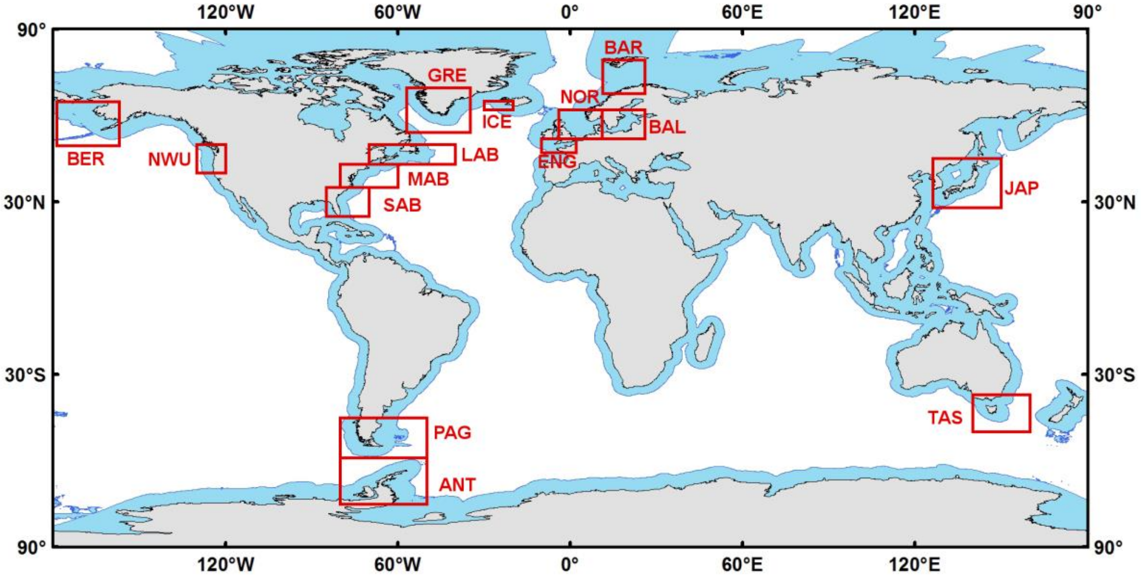

Supplementary Figure 5: Limits and code names of the 15 regions used for our analysis.

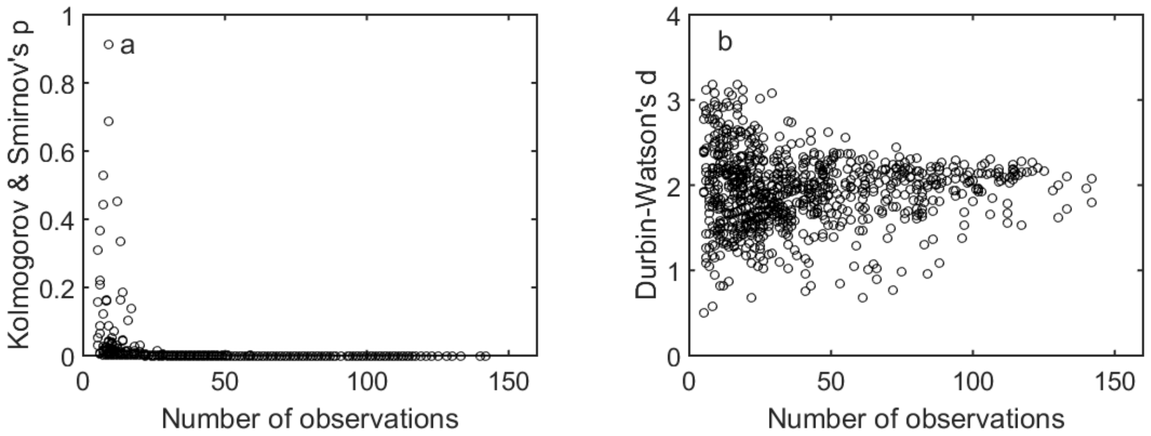

Supplementary Figure 6: Results of the Kolmogorov and Smirnov test (a) and Durbin and Watson's test (b) performed on the residuals of all time-series.

Supplementary Table 1: Number of cells displaying a statistically significant rate of change in ΔpCO_2 using an F-test with $p<0.05$ or $p<0.1$. The percentages and number of cells (n) are provided for cells displaying different $d(\Delta pCO_2)/dt$ during the winter season and the whole year.

Winter			
$d(\Delta pCO_2)/dt$	n	$p < 0.05$	$p < 0.1$
> 0.5	495	325 (66%)	355 (71%)
0.5 < > -0.5	246	27 (11%)	63 (13%)
-0.5 >	84	22 (26%)	34 (40%)
All year			
$d(\Delta pCO_2)/dt$	n	$p < 0.05$	$p < 0.1$
> 0.5	1489	574 (39%)	758 (51%)
0.5 < > -0.5	939	36 (4%)	63 (7%)
-0.5 >	1293	201 (24%)	273 (32%)

References:

1 Laruelle, G. G., Landschützer, P., Gruber, N., Tison, J.-L., Delille, B., & Regnier, P. Global high resolution monthly pCO_2 climatology for the coastal ocean derived from neural network interpolation, Biogeosciences, 14, 4545-4561, doi:10.5194/bg-14-4545-2017 (2017).
